# BET Family Protein BRD4: An Emerging Actor in NFκB Signaling in Inflammation and Cancer

**DOI:** 10.3390/biomedicines6010016

**Published:** 2018-02-06

**Authors:** Azadeh Hajmirza, Anouk Emadali, Arnaud Gauthier, Olivier Casasnovas, Rémy Gressin, Mary B. Callanan

**Affiliations:** 1 INSERM U1209, CNRS UMR 5309, Institute for Advanced Biosciences, Université de Grenoble-Alpes, F-38042 Grenoble, France; azadeh.hajmirza@univ-grenoble-alpes.fr (A.H.); anouk.emadali@univ-grenoble-alpes.fr (A.E.); agauthier@chu-grenoble.fr (A.G.); 2 Pôle Recherche, Grenoble-Alpes University Hospital, F-38043 Grenoble, France; 3 Département d’Hématologie Clinique, Dijon University Hospital, F-21000 Dijon, France; olivier.casasnovas@chu-dijon.fr; 4 Département d’Hématologie Clinique, Grenoble-Alpes University Hospital, F-38043 Grenoble, France; RGressin@chu-grenoble.fr; 5 Centre for Innovation in Cancer Genetics and Epigenetics, Dijon University Hospital, F-21000 Dijon, France

**Keywords:** NFκB, BET inhibition, transcription, chromatin looping, acetylation B cell non-Hodgkin lymphoma

## Abstract

NFκB (Nuclear Factor-*κ*-light-chain-enhancer of activated B cells) signaling elicits global transcriptional changes by activating cognate promoters and through genome-wide remodeling of cognate regulatory elements called “super enhancers”. BET (Bromodomain and Extra-Terminal domain) protein family inhibitor studies have implicated BET protein member BRD4 and possibly other BET proteins in NFκB-dependent promoter and super-enhancer modulation. Members of the BET protein family are known to bind acetylated chromatin to facilitate access by transcriptional regulators to chromatin, as well as to assist the activity of transcription elongation complexes via CDK9/pTEFb. BET family member BRD4 has been shown to bind non-histone proteins and modulate their activity. One such protein is RELA, the NFκB co-activator. Specifically, BRD4 binds acetylated RELA, which increases its transcriptional transactivation activity and stability in the nucleus. In aggregate, this establishes an intimate link between NFκB and BET signaling, at least via BRD4. The present review provides a brief overview of the structure and function of BET family proteins and then examines the connections between NFκB and BRD4 signaling, using the inflammatory response and cancer cell signaling as study models. We also discuss the potential of BET inhibitors for relief of aberrant NFκB signaling in cancer, focusing on non-histone, acetyl-lysine binding functions.

## 1. Introduction

Epigenetic signaling refers to the chromatin-dependent mechanisms that directly or indirectly control genome activity. Essentially, this refers to the chemical modifications on DNA or chromatin (histone proteins) that, together with topological organization of the chromatin fiber in the nucleus, regulate chromatin compaction. This allows the formation of functionally distinct, dynamically reversible chromatin states called euchromatin and heterochromatin, respectively [1]. The former is characterized by loosely packed nucleosomes, while in heterochromatin nucleosomes are densely packed. Nucleosomes are the basic functional unit of chromatin and are comprised of an octamer of the core histones, H2A, H2B, H3, and H4 (two copies of each). DNA methylation and post-translational histone modifications allow regulation of genome function by the modulation of chromatin compaction- and thereby access to DNA by transcription, replication, and repair factors- by serving as docking platforms for specific chromatin-associated signaling complexes [1]. Enzymes that mediate chemical modifications of DNA or chromatin are referred to as “writers” of epigenetic information, while those proteins that dock or erase these chemical modifications are referred to as “readers” and “erasers” or epigenetic information, respectively [1].

Bromodomain and extra-terminal domain (BET) proteins constitute a novel class of epigenetic “readers” that is involved in the control of genome activity through the ability to bind acetylated lysine residues in both histone and non-histone proteins, including transcription factors. The mechanism by which BET proteins are recruited to acetylated lysine is discussed below. How this links to transcriptional control by NFκB signaling is reviewed.

**Figure 1:**
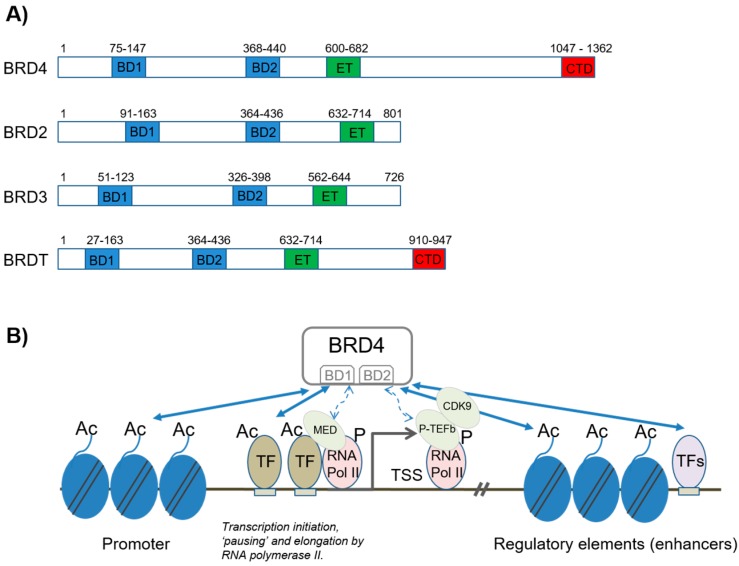
Schematic of domain organization of BET (bromodomain and extra-terminal domain) family proteins and the function of BRD4 in the regulation of promoter and enhancer activity. (**A**) Domain organization of human BET family members BRD4, BRD2, BRD3, and BRDT, as indicated. BET proteins contain two bromodomains (BD1 and BD2, respectively) and an extra-terminal domain (ET). BRD4 (long form) contains a carboxyterminal domain (CTD) that is not present in the other BET family members; (**B**) Schematic representation of the functions of BET family member BRD4 in the regulation of promoter and enhancer function (includes “super-enhancers”; see text for details). Through its BD1 and BD2 domains, BRD4 binds to acetylated lysines (Ac) in histones or transcription factors (TF). The binding of acetylated histones by BRD4, at transcription start sites (TSS), mediates transcriptional co-activation and elongation via RNA polymerase II (RNA pol II) and Mediator (Med) and pTEFb signaling complexes, respectively (see text for details). BRD4 can also bind acetylated lysines in histones or TF in enhancer elements, thereby contributing to long-range control of gene activity (see text for details). TF binding sites are depicted as horizontal rectangles.

BET proteins have emerged as key regulators of transcriptional control in development and cellular differentiation and have been identified as critical actors of disordered transcription in transformed cells, thereby rendering them hypersensitive to small molecule inhibition of BET protein activity [2,3]. Mechanisms of action are complex but rely on the capacity of these proteins to bind acetylated histone and non-histone proteins via their double bromodomains (Figure 1) [2].

## 2. Structure and Function of BET Proteins

The BET family of proteins comprises BRD2, BRD3, BRD4 (ubiquitously expressed), and BRDT (testis-specific expression). BET proteins are characterized by the presence of two conserved *N*-terminal bromodomains (BD1 and BD2) and a C-terminal “extra-terminal” domain (Figure 1). The bromodomain structure contains four alpha helices separated by a variable loop region that together allow the formation of a hydrophobic cavity that recognizes acetyl-lysine residues [4,5]. Structural data have established that acetylated lysine is recognized in this central hydrophobic pocket, by anchoring to a conserved asparagine residue. The BET bromodomain proteins can bind to two acetylated lysine histone marks that are simultaneously recognized by the same bromodomain module [5]. This property is shared by all members of the BET subclass of bromodomains. High-resolution co-crystal structures showed that the first acetylated lysine mark of histone H4 docks directly onto the conserved asparagine (Asn140 in the first bromodomain of BRD4). A network of hydrogen bonds within the acetyl-lysine binding cavity link to the second acetylated lysine mark thus stabilizing the peptide complex [4,5]. The largely hydrophobic nature of the central acetylated lysine binding pocket of the bromodomain, which is necessary to accommodate the charge-neutralized acetylated lysine, and the comparably weak interaction with its target sequences make these modules particularly attractive for the development of inhibitors targeting this protein–protein interaction. The interaction of BET proteins with mono-acetylated lysine seems to be of weak affinity compared to the interactions that occur at multiple closely spaced acetylated lysines [4,5].

As well as interacting with acetylated lysine in histones, BET proteins also interact with members of the transcription elongation complex and with other transcription factors. In the latter case, this can be through lysine acetylation-dependent or -independent mechanisms. As such, BET proteins are key “readers” of epigenetic information in both normal and transformed cells. The interaction of BDs at acetylated chromatin either at gene promoters or in long range *cis* regulatory elements called enhancers allows chromatin-dependent signaling to connect to transcription regulation [6].

### 2.1. BRD4 in Transcriptional Regulation by NFκB

BRD4 is a particularly well-studied member of the BET protein family. BRD4 contains two bromodomains associated to an extra-terminal domain (Figure 1A). As explained above, the BD domains allow BRD4 to interact with acetylated lysine in histone or non-histone proteins. How this property is involved in transcriptional regulation is discussed. New findings relating to BET proteins and pro-inflammatory- or cancer cell-specific NFκB signaling [7] are also reviewed.

#### 2.1.1. Transcription Initiation and Elongation

BRD4 participates in the activation and elongation of transcription via interactions with transcription initiation and elongation complexes Mediator and pTEFb (positive transcription elongation factor B), respectively. The pTEFb complex is composed of the cyclin-dependent kinase, CDK9, and a regulatory subunit, Cyclin T1 or T2. The kinase activity of CDK9 inhibits negative regulators of RNA polymerase II activity while stimulating its elongation activity by phosphorylation [8]. At least two different regions of BRD4 interact directly with pTEFb. The C-terminal region interacts with Cyclin T1 and CDK9 and the BD2 region interacts with the acetylated region of Cyclin T1. The BRD4/pTEFb interaction plays a central role in the rapid initiation of transcription after the exit from mitosis [8].

The extra-terminal (ET) domain is involved in transcriptional regulation through interactions with histone modifiers such as JMJD6 (jmjC domain-containing protein 6), an arginine demethylase, and NSD3, a lysine methyltransferase [9,10]. Furthermore, the ET domain can associate with ATP-dependent chromatin remodelers such as the SWI-SNF and CHD2 [10]. These interactions are thought to allow BRD4 to remodel chromatin locally, although the regulatory significance of these events is not well understood. One possibility is that this allows the release of paused RNA pol II activity [9].

NFκB-dependent transcriptional control is regulated at multiple levels, including cytoplasmic signaling events leading to the nuclear translocation of NFκB, the binding of nuclear NFκB to various transcriptional factors or regulators, and the post-transcriptional modifications of histones and NFκB itself [7]. Within the nucleus, NFκB recognizes the cognate NFκB sites on the enhancer or promoter regions of its target genes and directs the binding of co-regulators to form the transcriptional machinery for target gene expression. In the setting of NFκB signaling, it has been found that pTEFb can be recruited by BRD4 to NFκB-dependent acetylated histones—a mechanism that is crucial for the transcription of primary response genes [6], and possibly pathological NFκB signaling in cancer cells, although the latter has not been investigated in any detail as yet.

#### 2.1.2. Enhancer Regulation by BRD4 and Its Role in NFκB Signaling

The genome-wide distribution of BRD4 has been studied by chromatin immunoprecipitation and deep sequencing (ChIP-seq). These experiments have shown that BRD4 binds multiple promoters as well as intergenic regions, particularly enhancer sequences. The Mediator complex (MED), composed of 26 subunits in mammals, plays a key role in transcription initiation and elongation downstream of numerous signaling cascades as well as in the functional regulation of enhancer elements. BRD4 and MED have been found to co-occupy subsets of enhancers called “super-enhancers” [11], which are large enhancer regions that stimulate the transcription of growth-promoting and lineage-specific survival genes [6]. Super-enhancers are also co-enriched for histone H3 acetylated at lysine 27. Supporting a functional interaction of BRD4 and MED at super-enhancers, BET bromodomain inhibition releases the mediator complex from select *cis*-regulatory elements, at least in leukemia cells [12]. It is interesting to note that MED complex activity at super-enhancers involves reversible association with a subunit containing the cyclin dependent kinase CDK8 and the cofactors CCNC (CYCLIN C), MED12, and MED13. Mutations in the gene encoding MED12 have been described in chronic lymphocytic leukemia [13]. Furthermore, the MED complex contains both activating and inhibiting CDKs, the latter of which appear to constrain tumor suppressor and lineage identity gene-associated super-enhancers, which raises interest in combining BET and MED complex negative regulatory CDK inhibitors for anti-cancer treatment [14].

Numerous oncogenes have been shown to be under the control of super-enhancer elements in various cancer types. Remarkable examples are the deregulation of MYC in B cell non-Hodgkin lymphoma and multiple myeloma [15,16], EVI1 in acute myeloid leukemia with the inversion of chromosome 3q [17], and mutational processes that are predicted to alter super-enhancer activity in breast cancer [18]. The recruitment of BRD4 to enhancer regions seems to depend, at least in part, on the activity of specific transcription factors and on histone acetyl-transferases such as p300/CBP [19]. The recruitment of BRD4 is essential to the activity of numerous hematopoietic transcription factors such as PU.1, FLI1, ERG, C/EBPα, C/EBPβ, and MYB [19] and to the activity of NFκB at cognate enhancers, downstream of inflammatory responses in endothelial cells and macrophages [20]. Although the precise mechanism for BRD4 co-recruitment with the NFκB subunit RELA/p65 to pro-inflammatory genes is not yet deciphered, it may relate to both BRD4-dependent histone lysine acetylation docking as well as to the ability of BRD4 to bind acetylated p65/RELA, as discussed in detail below [21].

NFκB-dependent enhancer remodeling during pro-inflammatory responses has been shown to implicate the synthesis of enhancer RNA (eRNA) and local chromatin acetylation, followed by progressive H3 lysine 4 mono and di-methylation [22]. This process requires composite binding with other tissue-specific transcription factors. eRNAs belong to the non-coding RNAs and their transcripts are directed by enhancers, which is thought to favor the spatial repositioning (‘chromatin looping’) of distal enhancers close to their cognate promoters via RNA pol II. Thus, NFκB acts as a key modulator of de novo enhancer remodeling in the setting of pro-inflammatory signaling by modulating eRNA transcription [23].

In keeping with the above findings, BET inhibitors effectively suppress inflammatory responses mediated by NFκB, in particular at super-enhancers [20]. Likewise, the NFκB pathway is activated by LPS (lipopolysaccharide), and the pan-BET inhibitor I-BET762 has been shown to prevent or diminish the incidence of death in mice given lethal doses of lipopolysaccharide [24].

#### 2.1.3. BRD4 Interaction with Acetylated RELA/p65; Impact on NFκB Signaling and Sensitivity to BET Inhibitors

**Figure 2:**
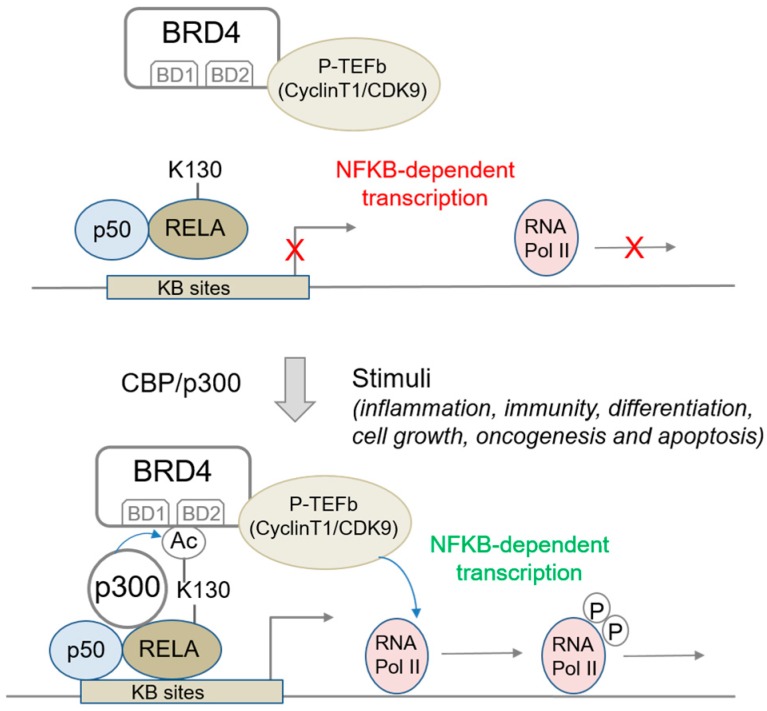
Schematic model for the binding of BRD4 to acetylated lysine-310 of RELA/p65 and the role of this interaction in the transcriptional activation of NFκB. Stimulus-dependent acetylation of RELA at lysine-310 by p300 induces the recruitment of BRD4 to the promoter via its bromodomains. BRD4 further activates CDK9 to phosphorylate the CTD (*C*-terminal domain) of RNA polymerase II and to facilitate the RNA pol II-mediated transcription of NFκB target genes. The p50 factor is a processed form of the REL family member p105 (NFκB1) that heterodimerizes with RELA/p65 and that is required for its transcriptional activation functions.

BRD4/BET proteins have been shown to interact with acetylated lysines in non-histone proteins [6]. This facet of BRD4/BET activity has not been explored in detail in cancer, but is likely to underlie at least some of the clinical activity of BET inhibitors, in particular in relation to aberrant NFκB signaling. Indeed, BRD4 interacts with the NFκB subunit, p65/RELA, when the latter is acetylated at lysine 310 [21] (and references therein). Under NFκB activation conditions, this leads to the recruitment of pTEFb and stimulates the transcription of NFκB target genes [21], in a BRD4- and CDK9-dependent fashion. RELA/BRD4 co-recruitment to NFκB target promoters is observed under a variety of NFκB stimulation conditions and cell types, and is not observed at housekeeping genes, supporting a necessity for defined promoter features for this interaction. Mapping and amino acid exchange studies have identified RELA acetyl-lysine-310 as the key residue for this interaction. Indeed, lysine substitution by an arginine residue significantly reduces BRD4 co-recruitment to an NFκB target promoter, as measured by ChIP assays [21]. Importantly, this does not alter the recruitment of either CBP/p300 or non-acetylated RELA, while acetylated RELA recruitment is lost. Taken together, this points to a critical role for acetylated RELA lysine-310 in BRD4 recruitment at NFκB response promoters. The acetylation of RELA lysine 310 is mediated by p300/CBP (Figure 2).

Of note, both bromodomains of BRD4 are necessary for the interaction with lysine-310-acetylated RELA, as evidenced by pull down assays using bromodomain deletion mutants, and in vivo immunoprecipitation assays. Moreover, loss of this interaction results in the inability of BRD4 to co-activate NFκB-dependent transcription. Structural studies have confirmed this by showing that both BD1 and BD2 bind to acetyl-lysine-310 peptide, and that this occurs through the conserved Asn140 in BD1 and Asn433 in BD2, respectively [25]. Consistent with this result, the dual bromodomain inhibitor JQ1 blocks the interaction between BRD4 and acetylated RELA, coincident with the suppression of NFκB-induced transcriptional responses [25]. Treatment with the BET inhibitor JQ1 releases RELA for ubiquitination and proteasome-mediated degradation. RELA transcript levels are not affected. In contrast, the levels of a second subunit of the NFκB signaling complex that binds RELA, p50, are unaltered. In this regard, it is worth noting that MS417, a derivative of the triazolo-thienodiazepine compound class, also prevents BRD4 binding to acetylated NFκB, thus reducing the transcriptional activity of this factor [26].

Previous studies have shown that the RELA/BRD4 interaction is favored by the phosphorylation of RELA/p65 at serines 276 and 536, which allows recruitment of histone acetyl-transferase p300/CBP [27]. Interestingly, enhanced STAT3-dependent RELA phosphorylation has been shown to increase RELA acetylation by p300/CBP and has been implicated in constitutive NFκB activity in human and murine tumors [25,28].

Of note, the mono-methylation of RELA lysine 310 by SETD6 has been identified and shown to couple NFκB activity to that of histone methyltransferase EHMT1/GLP (Euchromatic histone-lysine *N*-methyltransferase 1) at chromatin, thereby leading to the tonic repression of NFκB signaling at target promoters [29]. RELA/p65 lysine 310 mono-methylation is blocked by activation-dependent phosphorylation at Ser311, by protein kinase C-ζ(PKC-ζ). How this impacts subsequent lysine 310 acetylation is not known in detail.

In aggregate, the above data underscore the feasibility and potential clinical interest of targeting BRD4 interactions with non-histone acetylated lysines in anti-cancer therapy. Targeting the BRD4/acetylated RELA axis should be of value in NFκB-dependent cancers, such as lymphoma, where aberrant NFκB signaling is frequent, including through genetic mechanisms [30].

## 3. BET Inhibition as an Anti-Cancer Therapy and Perspectives for Use in NFκB-Dependent Cancers

Numerous studies indicate that BET inhibition is an attractive therapeutic strategy in hematological and solid cancers. Single or dual agent therapy is showing promise pre-clinically [1,3,6,31,32]. Inhibitors are mostly small molecule inhibitors that are thought to mediate anti-cancer activity through the interruption of interactions between BET proteins and acetylated lysine in histones at promoters and enhancers. A number of these inhibitors are being tested in early phase clinical trials, in hematological and solid cancers, as either single agents or in combination with other treatments (Table 1; and https://clinicaltrials.gov/ for further details on BET inhibitor trials). Although published clinical data remain sparse, one BET inhibitor, OTX015, has shown efficacy in patients with refractory or relapsed hematological cancers [33,34].

In this review, by focusing on BRD4-mediated regulation of NFκB signaling via acetylated RELA, we make a case for a second avenue of investigation for the development of therapeutic strategies utilizing BET inhibitors. Specifically, we propose that BRD4-dependent signaling via non-histone acetyl-lysine interactions will be of interest. Experimental data indicate that small molecule inhibition of the RELA-BRD4 interaction offers promise for the disruption of pathological NFκB signaling in cancer, a process which has been attributed to the inability of cancer cells to homeostatically control NFκB function [35]. Functional and chemical genomics approaches should allow progress in the identification and/or design of additional agents for pre-clinical testing in NFκB-dependent cancers such as lymphoma.

## Figures and Tables

**Table 1:** Summary of BET inhibitor clinical trials (open Dec. 2017).

Compound	Company	Indications	Phases	Completion Date	NCT Number
FT-1101	Forma Therapeutics, Inc. (Watertown, MA, USA)	Acute Myeloid Leukemia/Acute Myelogenous Leukemia/Myelodysplastic Syndrome	Phase 1	August 2018	NCT02543879
RO6870810 *	Hoffmann-La Roche (Basel, Switzerland)	Multiple Myeloma	Phase 1	15 January 2020	NCT03068351
CPI-0610	Constellation Pharmaceuticals (Cambridge, MA, USA)	Lymphoma	Phase 1	July 2018	NCT01949883
		Multiple Myeloma	Phase 1	March 2019	NCT02157636
		Acute Myeloid Leukemia/Myelodysplastic Syndrome (MDS)/ Myelodysplastic/Myeloproliferative Neoplasm, Unclassifiable/Myelofibrosis	Phase 1	January 2019	NCT02158858
		Peripheral Nerve Tumors	Phase 2	March 2020	NCT02986919
GSK525762	GSK (Brentford, UK)	Cancer	Phase 1	24 February 2020	NCT01943851
		Carcinoma, Midline	Phase 1	9 September 2019	NCT01587703
ZEN003694 **	Zenith Epigenetics (San Francisco, CA, USA)	Metastatic Castration-Resistant Prostate Cancer	Phase 1	April 2018	NCT02711956
BMS-986158	Bristol-Myers Squibb (New York, NY, USA)	Multiple Indications Cancer	Phase 1/Phase 2	17 December 2018	NCT02419417

* Alone or in combination with Daratumumab; ** in combination with Enzalutamide.
